# Choline Kinase Alpha Is a Novel Transcriptional Target of the Brg1 in Hepatocyte: Implication in Liver Regeneration

**DOI:** 10.3389/fcell.2021.705302

**Published:** 2021-08-06

**Authors:** Ming Kong, Wenhui Dong, Huihui Xu, Zhiwen Fan, Xiulian Miao, Yan Guo, Chengping Li, Qing Ye, Yutong Wang, Yong Xu

**Affiliations:** ^1^Key Laboratory of Targeted Intervention of Cardiovascular Disease, Department of Pathophysiology, Collaborative Innovation Center for Cardiovascular Translational Medicine, Nanjing Medical University, Nanjing, China; ^2^Institute of Biomedical Research, Liaocheng University, Liaocheng, China; ^3^Department of Pathology, Nanjing Drum Tower Hospital Affiliated with Nanjing University School of Medicine, Nanjing, China; ^4^College of Life Sciences, Liaocheng University, Liaocheng, China; ^5^Division of Life Sciences and Medicine, Department of Pathology, The First Affiliated Hospital, University of Science and Technology of China, Hefei, China; ^6^Division of Life Sciences and Medicine, Intelligent Pathology Institute, University of Science and Technology of China, Hefei, China; ^7^Department of Cell Biology, The Municipal Laboratory of Liver Protection and Regulation of Regeneration, School of Basic Medical Sciences, Beijing, China

**Keywords:** transcriptional regulation, choline kinase, transcription factor, epigenetics, chromatin remodeling protein, hepatocyte, liver regeneration

## Abstract

Liver regeneration is a key compensatory process in response to liver injury serving to contain damages and to rescue liver functions. Hepatocytes, having temporarily exited the cell cycle after embryogenesis, resume proliferation to regenerate the injured liver parenchyma. In the present study we investigated the transcriptional regulation of choline kinase alpha (Chka) in hepatocytes in the context of liver regeneration. We report that Chka expression was significantly up-regulated in the regenerating livers in the partial hepatectomy (PHx) model and the acetaminophen (APAP) injection model. In addition, treatment with hepatocyte growth factor (HGF), a strong pro-proliferative cue, stimulated Chka expression in primary hepatocytes. Chka depletion attenuated HGF-induced proliferation of hepatocytes as evidenced by quantitative PCR and Western blotting measurements of pro-proliferative genes as well as EdU incorporation into replicating DNA. Of interest, deletion of Brahma-related gene 1 (Brg1), a chromatin remodeling protein, attenuated Chka induction in the regenerating livers in mice and in cultured hepatocytes. Further analysis revealed that Brg1 interacted with hypoxia-inducible factor 1 alpha (HIF-1α) to directly bind to the Chka promoter and activate Chka transcription. Finally, examination of human acute liver failure (ALF) specimens identified a positive correlation between Chka expression and Brg1 expression. In conclusion, our data suggest that Brg1-dependent trans-activation of Chka expression may contribute to liver regeneration.

## Introduction

Acute or chronic loss of liver function following a wide range of injuries disrupts internal homeostasis and leads to liver failure with life-threatening consequences (Arroyo et al., 2020). Liver regeneration serves as a crucial compensatory mechanism by restoring the damaged liver mass and normalizing liver function (Michalopoulos, 2017). Having temporarily exited the cell cycle, hepatocytes could resume proliferation to replenish the loss of liver parenchyma and alleviate the decline of liver function. Therefore, the robustness with which existing hepatocytes can proliferate is observed to correlate with the prognosis of patients with end-stage liver diseases (Delhaye et al., 1999; Lanthier et al., 2015). Proliferation of quiescent hepatocyte and hence liver regeneration are guided by a complex signaling network. Hepatocyte growth factor (HGF) is one of the best characterized pro-proliferative cues residing at the center of this network. Deletion of HGF (Phaneuf et al., 2004) or its receptor (c-Met) (Borowiak et al., 2004) impairs liver regeneration in mice. On the contrary, recombinant HGF appears to confer protection against liver failure by boosting liver regeneration in mice (Kosai et al., 1999) and in humans (Cui et al., 2008). In accordance, serum HGF levels have been proposed as a prognostic marker for patients with liver failure (Shiota et al., 1995; Ido and Tsubouchi, 2009). When stimulated by HGF, hepatocytes undergo marked transcriptomic alterations with multiple clusters of genes being up- or down-regulated (Kaposi-Novak et al., 2006). Regulatory mechanisms underlying these transcription events and the implications in liver regeneration have yet to be fully understood.

Choline kinases catalyze the biosynthesis of phosphatidylcholine, a key component of cellular membranes (McMaster, 2018). Two isoforms, choline kinase alpha (Chka) and Chkb, have been identified with Chka being the predominant isoform in mammals (Aoyama et al., 2004). The essentiality of Chka in choline metabolism is evidenced by the observation that mice homozygous for Chka deletion die prematurely during embryogenesis (Wu et al., 2008). Recent studies have shown that over-expression/hyperactivation of Chka, but not Chkb, is frequently observed in and associated with neoplastic transformation of multiple cell lineages (Gallego-Ortega et al., 2011). For instance, Lin et al. (2017) have reported that Chka promotes aberrant proliferation of hepatocellular carcinoma (HCC) cells by mediating the crosstalk between the EGF signaling and the mTOR signaling. Chka levels have also been shown to correlate positively with malignant expansion of liver tumors and inversely with patient outcome (Kwee et al., 2012; Carrillo-Reixach et al., 2020). Of interest, concurrent high expression of Chka and HGF has been noted in the HCC patients (Mok et al., 2016). It remains undermined whether Chka expression can be regulated by HGF in hepatocytes.

Brahma-related gene 1 (Brg1) is a chromatin remodeling protein playing diverse roles in orchestrating both physiological and pathophysiological events in the liver. Previously we have reported that Brg1 deficiency attenuates diet-induced non-alcoholic steatohepatitis, concanavalin A-induced fulminant hepatitis, and lipopolysaccharide-induced acute liver injury in mice by regulating the transcription of pro-lipogenic genes, chemokine nephronectin, and pattern recognition receptor TLR4, respectively (Fan et al., 2020; Dong et al., 2021; Hong et al., 2021). On the other hand, Brg1 deletion in hepatocytes results in impairment of liver regeneration owing to dampened induction of pro-proliferative genes downstream of the Wnt-β-catenin signaling (Li et al., 2019). In the present study we investigated the transcriptional mechanism whereby Chka expression is regulated in hepatocyte in the context of liver regeneration. Our data suggest that Brg1 forms a transcriptional complex with hypoxia inducible factor to activate Chka transcription in hepatocytes.

## Materials and Methods

### Animals

All animal protocols were reviewed and approved the intramural Ethics Committee on Humane Treatment of Laboratory Animals of Nanjing Medical University. The mice were maintained in an SPF environment with 12 h light/dark cycles and libitum access to food and water. Hepatocyte conditional Brg1 knockout (Brg1^LKO^) mice have been described previously (Kong et al., 2021). Liver regeneration by the partial hepatectomy (PHx) procedure or intraperitoneal injection of acetaminophen as previously described (Li et al., 2019).

### Cells, Transient Transfection, and Reporter Assay

Primary murine hepatocytes were isolated as previously described (Fang et al., 2020; Wang et al., 2020c). Mouse recombinant HGF was purchased from R&D. Brg1 expression construct (Li et al., 2020c; Sun et al., 2020) and *Chka* promoter-luciferase constructs (Glunde et al., 2008) have been previously described. Small interfering RNAs were purchased from GenePharma. Transient transfections were performed with Lipofectamine 2000. Luciferase activities were assayed 24–48 h after transfection using a luciferase reporter assay system (Promega) as previously described (Li et al., 2020b; Mao et al., 2020).

### Protein Extraction and Western Blot

Whole cell lysates were obtained by re-suspending cell pellets in RIPA buffer (50 mM Tris pH 7.4, 150 mM NaCl, 1% Triton X-100) with freshly added protease inhibitor (Roche) as previously described (Chen et al.,2020b,c). Western blot analyses were performed with anti-Chka (Proteintech, 13520-1), anti-Brg1 (Abcam, Ab110641), and anti-β-actin (Sigma, A1978). For densitometrical quantification, densities of target proteins were normalized to those of β-actin. Data are expressed as relative protein levels compared to the control group which is arbitrarily set as 1.

### RNA Isolation and Real-Time PCR

RNA was extracted with the RNeasy RNA isolation kit (Qiagen). Reverse transcriptase reactions were performed using a SuperScript First-strand Synthesis System (Invitrogen) as previously described (Chen et al.,2020a,b,c,2021; Dong et al., 2020, 2021; Fan et al., 2020; Li et al.,2020a,b,c; Lv et al., 2020; Mao et al., 2020; Sun et al., 2020; Wu T. et al., 2020; Wu X. et al., 2020; Yang et al., 2020; Hong et al., 2021; Kong et al., 2021; Liu et al.,2021a,b; Zhang et al., 2021). Real-time PCR reactions were performed on an ABI Prism 7500 system with the following primers: *Chka*, GGGTGGTCTCAGTAACATGCT and GAACCCTGGACTCACCATCTT; *Ccna2*, TGGATGGC AGTTTTGAATCACC and CCCTAAGGTACGTGTGAATGTC; *Ccnd1*, GCGTACCCTGACACCAATCTC and ACTTGA AGTAAGATACGGAGGGC; *Cdk1*, CTTTGTCCGAGAGTTT CAGCC and AGTTCCGGGTAGACATCTTTGT; and *Ccnb1*, CAATTATCGGAAGTGTCGGATCA and CTGGTGAACGACT GAACTCCC. Ct values of target genes were normalized to the Ct values of housekeeping control gene (18s, 5′-CGC GGTTCTATTTTGTTGGT-3′ and 5′-TCGTCTTCGAAACTC CGACT-3′ for both human and mouse genes) using the ΔΔCt method and expressed as relative mRNA expression levels compared to the control group which is arbitrarily set as 1.

### Chromatin Immunoprecipitation

Chromatin immunoprecipitation (ChIP) assays were performed essentially as described before (Coarfa et al., 2020; Hu et al., 2020; Jehanno et al., 2020; Maity et al., 2020; Mallik et al., 2020; Moon et al., 2020; Shen et al., 2020; Wang et al.,2020a,b; Zhang et al., 2020; Zhao et al., 2020; Marti et al., 2021; Peng et al., 2021; Rashid et al., 2021). In brief, chromatin in control and treated cells were cross-linked with 1% formaldehyde. Cells were incubated in lysis buffer (150 mM NaCl, 25 mM Tris pH 7.5, 1% Triton X-100, 0.1% SDS, 0.5% deoxycholate) supplemented with protease inhibitor tablet and PMSF. DNA was fragmented into ∼200 bp pieces using a Branson 250 sonicator. Aliquots of lysates containing 200 μg of protein were used for each immunoprecipitation reaction with anti-Brg1 (Abcam, Ab110641), anti-hypoxia-inducible factor 1 alpha (HIF-1α) (Cell Signaling Techology, 14179), or pre-immune IgG. Precipitated DNA was amplified with the following primers: primer #1: 5′-ATATTACCAATCAGCGGCGAGC-3′ and 5′-ACTGCTATAGGGGGCGC-3′; primer #2, 5′-AAAAGCATGT GCCATTATGC-3′ and 5′-ATTCCTGGCTTTGTGGATGC-3′.

### Histology

Histological analyses were performed essentially as described before (Wu T. et al., 2020; Wu X. et al., 2020). Paraffin sections were stained with were blocked with 10% normal goat serum for 1 h at room temperature and then incubated with anti-Chka (Proteinch, 1:200) or anti-PCNA (Abcam, 1:200) antibodies. Staining was visualized by incubation with anti-rabbit secondary antibody and developed with a streptavidin-horseradish peroxidase kit (Pierce) for 20 min. Pictures were taken using an Olympus IX-70 microscope. Quantifications were performed with Image J.

### Human ALF Specimens

Liver biopsies were collected from patients with acute liver failure (ALF) referring to Nanjing Drum Tower Hospital (8 total). Written informed consent was obtained from subjects or families of liver donors. All procedures that involved human samples were approved by the Ethics Committee of the Nanjing Drum Tower Hospital and adhered to the principles outlined in the Declaration of Helsinki. Paraffin sections were stained with indicated antibodies.

### Statistical Analysis

One-way ANOVA with *post hoc* Scheffé analyses were performed by SPSS software (IBM SPSS v18.0, Chicago, IL, United States). Unless otherwise specified, values of *p* < 0.05 were considered statistically significant.

## Results

### Chka Up-Regulation Parallels Liver Regeneration *in vivo* and *in vitro*

**FIGURE 1:**
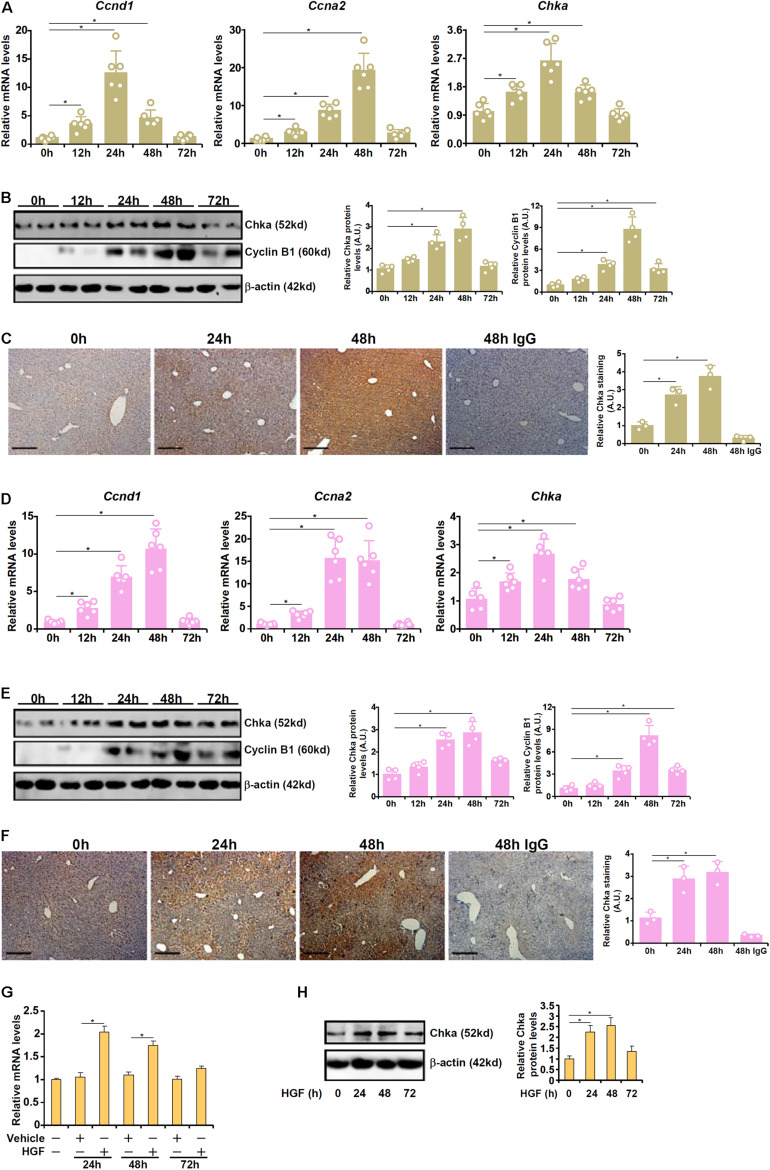
Choline kinase alpha expression levels are up-regulated in models of liver regeneration *in vivo* and *in vitro*. **(A–C)** C57B6/L mice were subjected to 2/3 PHx and sacrificed at indicated time points. Chka expression was examined by qPCR **(A)**, western blotting **(B)**, and immunohistochemical staining **(C)**. **(D–F)** C57B6/L mice were subjected to APAP injection (300 mg/kg) and sacrificed at indicated time points. Chka expression was examined by qPCR **(D)**, Western blotting **(E)**, and immunohistochemical staining **(F)**. **(G,H)** Primary hepatocytes were isolated from C57B6/L mice and treated with or without HGF. Chka expression levels were examined by qPCR and Western blotting. *p < 0.05.

We first profiled the expression of Chka in different animal and cell models of liver regeneration. In the first model in which PHx was performed in C57B6/L mice, it was observed that Chka expression, as measured by quantitative PCR (qPCR), started to go up as early as 12 h after the surgery; Chka expression peaked at 24 h and gradually declined until it reached basal levels at 72 h (Figure 1A). These changes coincided with the induction of pro-proliferative genes (*Ccnd1* encoding cyclin D1 and *Ccna2* encoding cyclin A2). Western blotting (Figure 1B) and immunohistochemical staining (Figure 1C) confirmed that Chka protein expression followed a similar trend. In the second model in which C57B6/L mice were injected with a single dose of acetaminophen to induce acute liver injury, liver regeneration, as judged by the induction of *Ccnd1* and *Ccna2*, was observed as early as 12 h after the injection (Figure 1D); Chka expression was similarly up-regulated trending closely with the progression of liver regeneration (Figures 1D–F). In a cell model in which primary murine hepatocytes were treated with the pro-proliferative growth factor HGF, we found that Chka expression was again up-regulated (Figures 1G,H). Taken together, these data suggest that Chka expression may correlate with liver regeneration *in vivo* and *in vitro*.

### Chka Is Essential for HGF-Induced Proliferation of Hepatocyte

**FIGURE 2:**
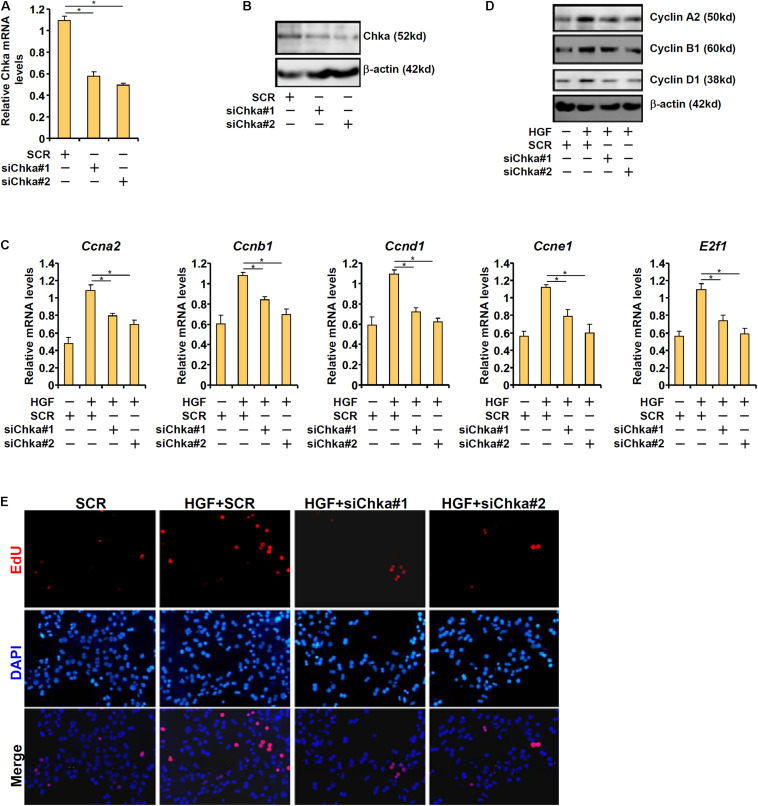
Choline kinase alpha is necessary for HGF-induced proliferation of hepatocyte. **(A,B)** Primary hepatocytes were transfected with indicated siRNAs and harvested 24 h after transfection. Chka expression was examined by qPCR and Western blotting. **(C–E)** Primary hepatocytes were transfected with indicated siRNAs followed by treatment with HGF for 24 h. Cell proliferation was examined by EdU staining. Pro-proliferative gene expression was examined by qPCR and Western blotting. *p < 0.05.

To provide support for a role of Chka in hepatocyte proliferation, endogenous Chka was depleted using two separate pairs of siRNAs, which potently and comparably down-regulated Chka expression in primary murine hepatocytes (Figures 2A,B). Chka knockdown significantly repressed the expression of several genes involved in cellular proliferation including cyclin A2 (Ccna2), cyclin B1 (Ccnb1), cyclin D1 (Ccnd1), cyclin E1 (Ccne1), and transcription factor E2F1 (E2f1) at both mRNA (Figure 2C) and protein (Figure 2D) levels. This observation is consistent with a previous report by Ramirez de Molina et al. (2008) in which Chka over-expression led to up-regulation of CCND1 in HEK293T cells. Another report by de la Cueva et al. (2013) in which Chka inhibition resulted in repression of E2F1 expression in colorectal cancer cells also appears to be in agreement with our observation.

We also measured the direct effect of Chka knockdown on hepatocyte proliferation by EdU incorporation. Immunofluorescence staining showed that Chka depletion markedly decreased HGF-induced EdU incorporation in primary hepatocytes indicative of suppression of proliferation (Figure 2E).

### Brg1 Deficiency Down-Regulates Chka Expression *in vivo* and *in vitro*

**FIGURE 3:**
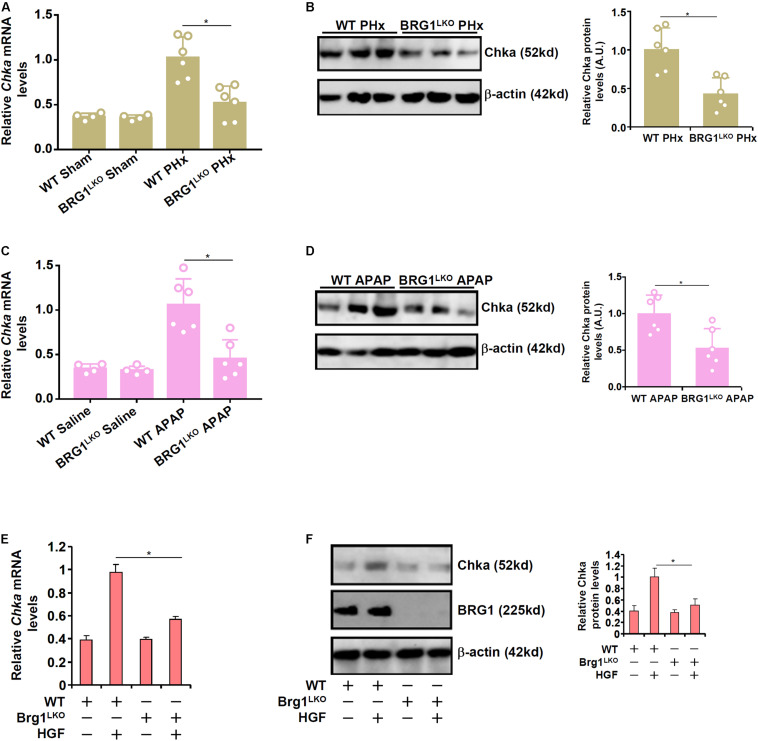
Brahma-related gene 1 deficiency attenuates Chka expression *in vivo* and *in vitro*. **(A,B)** WT and Brg1^LKO^ mice were subjected to 2/3 PHx and sacrificed 24 h after the surgery. Chka expression levels were examined by qPCR and Western blotting. **(C,D)** WT and Brg1^LKO^ mice were injected with APAP (300 mg/kg) and sacrificed 24 h after the injection. Chka expression levels were examined by qPCR and Western blotting. **(E,F)** Primary hepatocytes were isolated from WT and Brg1^LKO^ mice and treated with HGF for 24 h. Chka expression levels were examined by qPCR and Western blotting. *p < 0.05.

We have previously shown that the chromatin remodeling protein Brg1 contributes to liver regeneration by epigenetically activating the transcription of pro-proliferative genes (Li et al., 2019). Of interest, it was observed that although basal Chka expression was not significantly altered as a result of Brg1 deficiency in hepatocytes (Brg1^LKO^), there was a significant decrease in Chka expression in the Brg1^LKO^ livers following PHx compared to the WT livers (Figures 3A,B). Similarly, compared to the WT livers, the Brg1^LKO^ livers displayed reduced Chka expression following APAP injection but not saline injection (Figures 3C,D). Additionally, when primary hepatocytes were isolated from the Brg1^LKO^ mice and the WT mice and exposed to HGF treatment, Chka induction was much more modest in the Brg1^LKO^ cells than in the WT cells (Figures 3E,F).

### Brg1 Contributes to Chka Transcription

**FIGURE 4:**
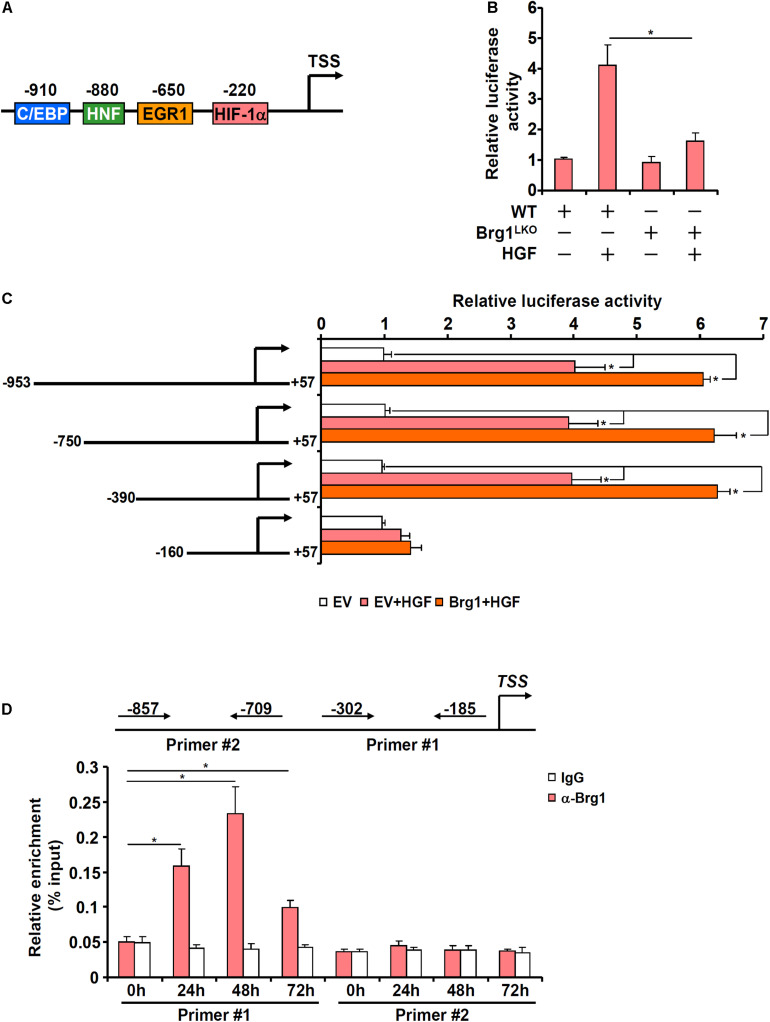
Brahma-related gene 1 contributes to Chka transcription in hepatocytes. **(A)** Schematic representation of the mouse Chka proximal promoter. TSS, transcription start site; HIF-1α, hypoxia induced factor 1; EGR1, early growth response 1; HNF, hepatocyte nuclear factor; C/EBP, CCAAT enhancer binding protein. **(B)** A Chka promoter-luciferase construct was transfected into primary hepatocytes isolated from WT and Brg1^LKO^ mice followed by HGF treatment. Luciferase activities were normalized by protein concentration and GFP fluorescence. **(C)** Chka promoter-luciferase constructs were transfected into primary hepatocytes with or without Brg1 followed by treatment with HGF. Luciferase activities were normalized by protein concentration and GFP fluorescence. **(D)** Primary murine hepatocytes were treated with HGF and harvested at indicated time points. ChIP assay was performed with anti-Brg1 or IgG. *p < 0.05.

In order to determine whether Brg1 regulates Chka expression at the transcriptional level, a Chka promoter-luciferase fusion construct (−953/+57, Figure 4A) was transfected into primary murine hepatocytes isolated from either the Brg1^LKO^ mice or the WT mice. HGF treatment significantly augmented the Chka promoter activity although the augmentation in the Brg1^LKO^ cells was not quite as strong as in the WT cells (Figure 4B). Therefore, it appeared that Brg1 might directly regulate Chka transcription. Further experimentation was carried out to determine the underlying mechanism. A series of truncated Chka promoter-luciferase fusion constructs were transfected into primary murine hepatocytes with or without Brg1 followed by HGF treatment. As shown in Figure 4C, Brg1 over-expression plus HGF treatment activated the Chka promoter-luciferase constructs until the deletion extended beyond −390 relative to the transcription start site, suggesting that Brg1 might occupy the region between −390 and −160. To verify whether Brg1 could indeed bind to the Chka promoter, ChIP assay was performed. The precipitated genomic DNA was amplified by two different pairs of primers: primer pair #1 (−302/−185) would generate an amplicon spanning the proximal Chka promoter region between −390 and −160 supposedly bound by Brg1 whereas primer pair #2 (−857/−709) would generate an amplicon spanning the more distal Chka promoter region. As shown in Figure 4D, HGF treatment elicited strong binding of Brg1 on the proximal Chka promoter but not the distal Chka promoter.

### HIF-1α Recruits Brg1 to Activate Chka Transcription in Hepatocytes

**FIGURE 5:**
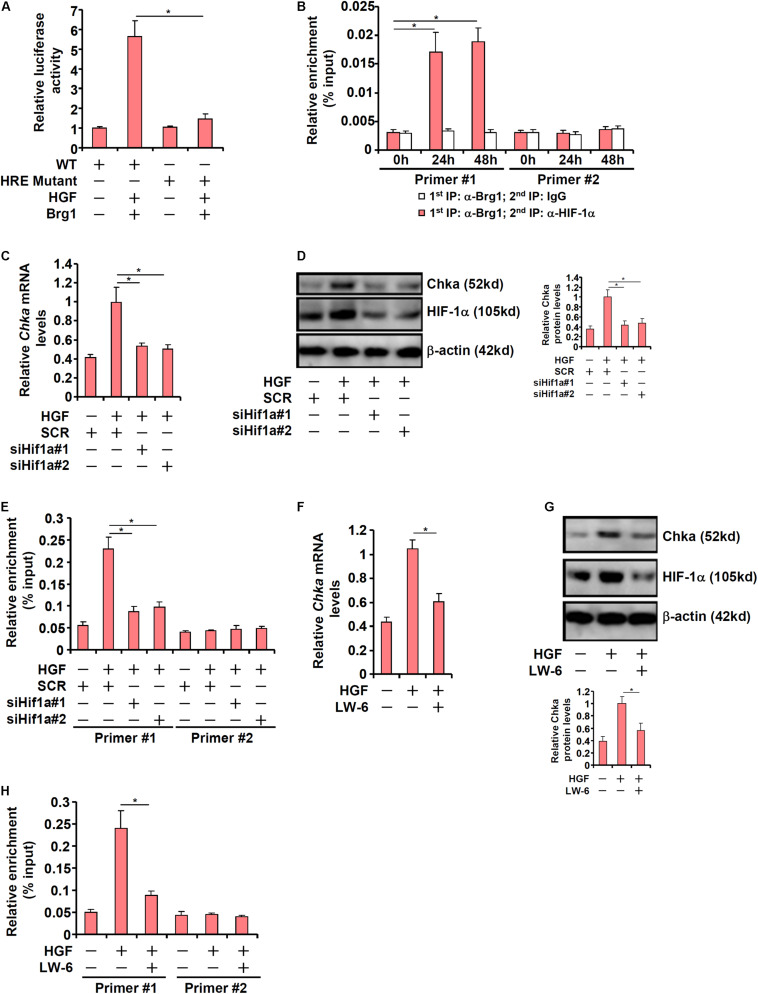
Hypoxia-inducible factor 1 alpha recruits Brg1 to activate Chka transcription in hepatocytes. **(A)** Wild type or mutant Chka promoter-luciferase construct was transfected into primary hepatocytes followed by HGF treatment. Luciferase activities were normalized by protein concentration and GFP fluorescence. **(B)** Primary murine hepatocytes were treated with HGF and harvested at indicated time points. Re-ChIP assay was performed with indicated antibodies. **(C–E)** Primary hepatocytes were transfected with indicated siRNAs followed by treatment with HGF. Chka expression was examined by qPCR and Western blotting. ChIP assay was performed with anti-Brg1. **(F–H)** Primary hepatocytes were treated with HGF in the presence or absence of LW-6 (5 μM). Chka expression was examined by qPCR and Western blotting. ChIP assay was performed with anti-Brg1. ChIP assay was performed with anti-Brg1. *p < 0.05.

Previously Glunde et al. (2008) have identified a conserved hypoxia response element (HRE) within the proximal Chka promoter. When this HRE was mutated within the context of the Chka promoter, Brg1 over-expression plus HGF treatment no longer triggered an activation (Figure 5A). ChIP-on-ChIP (Re-ChIP) assay indicated that HGF treatment promoted the interaction between Brg1 and HIF-1α on the Chka promoter (Figure 5B). In order to verify whether HIF-1α might be essential for Brg1 recruitment to the Chka promoter, the following experiments were performed. When endogenous HIF-1α was depleted with siRNAs, Chka induction by HGF was markedly down-regulated (Figures 5C,D). There was a simultaneous decrease in Brg1 occupancy on the Chka promoter as a result of HIF-1α depletion (Figure 5E). Alternatively, when HIF-1α was inhibited by the small-molecule compound LW-6, HGF-induced augmentation of Chka expression (Figures 5F,G) and Brg1 recruitment to the Chka promoter (Figure 5H) were significantly down-regulated. Together, these data identified a Brg1-HIF-1α interplay in mediating HGF-induced Chka transcription.

### Chka Expression Correlates With Brg1 Expression in ALF Patients

**FIGURE 6:**
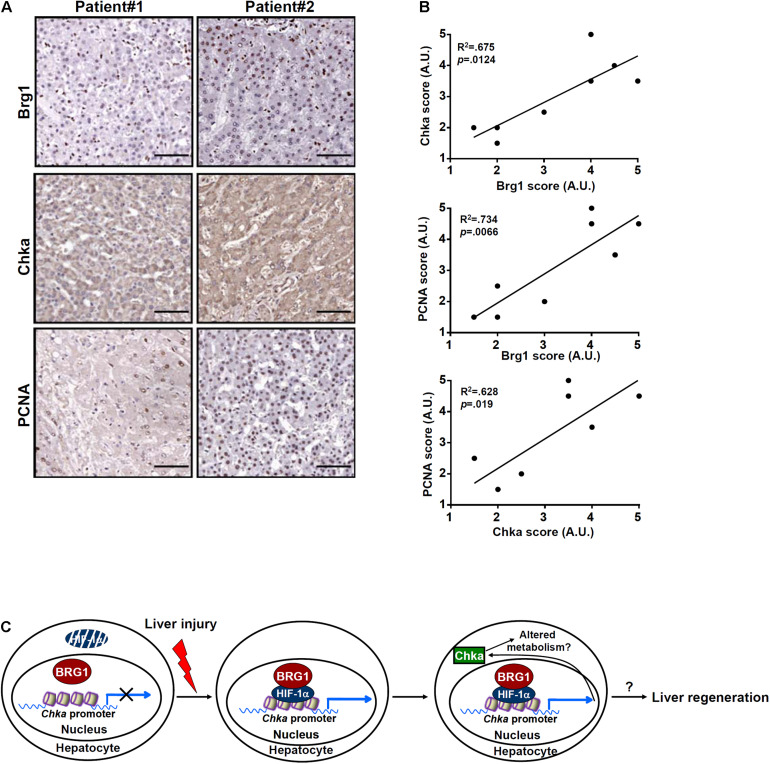
Choline kinase alpha expression correlates with Brg1 expression in ALF patients. **(A)** Paraffin sections of liver specimens collected from patients with acute liver failure were stained with anti-CHKA and anti-Brg1. *N* = 8 cases. **(B)** Linear regression was performed with GraphPad Prism. **(C)** A schematic model. Under physiological conditions, HIF-1α proteins are constantly degraded in hepatocytes presumably by the ubiquitin–proteasome system and Chka transcription is turned off. Exposed to injurious stimuli, HIF-1α proteins become stabilized and recruit BRG1 to activate Chka transcription. Elevated Chka levels may alter cellular metabolism and promote proliferation of hepatocytes eventually leading to liver regeneration.

Finally, we asked whether our proposed model that Brg1 regulates Chka expression in hepatocytes could be extrapolated to humans. To this end, paraffin-embedded sections of liver specimens from patients with ALF were stained for Chka expression and Brg1 expression by immunohistochemistry. As shown in Figure 6A, Chka expression was higher in the liver specimens with stronger staining for Brg1. Linear correlation analysis showed that there was a statistically significant (*p* < 0.05) positive correlation between Chka expression and Brg1 expression in the human livers (Figure 6B). In addition, when PCNA staining, generally considered to be indicative of hepatocyte proliferation and better prognosis for ALF patients, was performed and analyzed, it was found that both Brg1 expression and Chka expression appeared to be positively correlated with PCNA staining (Figure 6B), suggesting that the Brg1-Chka axis could potentially play a role in liver regeneration in humans.

## Discussion

We present evidence to show that Chka expression is synchronized with the regenerative response in hepatocytes both *in vivo* and *in vitro* (Figure 1). This is consistent with several previous reports pointing to a correlation between Chka expression and liver regeneration. Aoyama et al. (2007) have shown that Chka expression was up-regulated in the murine livers as early as 3 h after a single injection of carbon tetrachloride (CCl_4_). Because liver regeneration typically is initiated no earlier than 12 h after CCl_4_ injection in mice (McCracken et al., 2017), Chka induction clearly precedes full-scale hepatocyte proliferation. In another study Ming et al. (2017) report that Chka expression was induced by APAP injection in the liver peaking at 6 h. Whereas the kinetics of Chka induction by APAP in Ming et al.’s (2017) study is slightly ahead of our observation, both implicate Chka as an early response gene that presages the ensuing liver regeneration. Although Chka expression has not been examined in an exhaustive list of animal models, these data collectively do provide further insight on the potential role of Chka in liver regeneration.

Our data indicate that Chka depletion by siRNA attenuates the expression of pro-proliferative genes and dampens hepatocyte proliferation *in vitro*. The precise mechanism whereby Chka might contribute to hepatocyte proliferation remains to be determined. The best characterized role for Chka is the conversion of choline into phosphatidylcholine. Thus, Chka-deficient mice display augmented choline content and reduced phosphatidylcholine content in the liver (Wu et al., 2008). It has been observed that during rat liver regeneration following 70% PHx there is a decrease of hepatic choline content and a simultaneous increase of hepatic phosphatidylcholine content (Houweling et al., 1991). On the contrary, rats fed a choline-enriched diet exhibit delayed liver regeneration following PHx compared to the control diet-fed rats (Sesca et al., 1996). Coincidently, prolonged choline feeding leads to a decrease in the expression of c-Myc, a pro-proliferative proto-oncogene, in the rat livers (Sesca et al., 1996). Alternatively, Zhai et al. (2017) have shown that phosphatidylcholine can function as a ligand for liver receptor homolog 1 (LRH1), a nuclear receptor; liganded LRH1 forms a complex with β-catenin and enhances the transcription of β-catenin target genes involved in cell proliferation. Of interest, LRH1 depletion in zebrafish causes cell cycle arrest in the digestive organ. In addition, there is evidence to support a feedback circuit between phosphatidylcholine metabolism and SREBP1c activity: inhibition of phosphatidylcholine synthesis leads to SREBP1c hyperactivation (Walker et al., 2011). Because SREBP1c deficiency enhances liver regenerative response in mice (Peng et al., 2018), it is possible that Chka may contribute to liver regeneration by limiting SREBP1c activity. Due to the embryonic lethality of the Chka-null mice (Wu et al., 2008), it is not feasible to analyze the effect of Chka loss-of-function on liver regeneration *in vivo* at present time. Future studies should aim to generate a new transgenic animal model that restricts Chka deletion in the liver ideally in a temporally controllable manner to further examine the mechanism whereby Chka regulates liver regeneration.

We have previously shown that Brg1 is essential for liver regeneration in mice (Li et al., 2019). The new finding as summarized here shows that Brg1 interacts with HIF-1α to bind to the Chka promoter and activate Chka transcription. It remains to be determined whether Brg1 relies on Chka to exert the pro-proliferative effects in hepatocytes. An equally lingering but intriguing question is whether transcriptional mechanisms, other than the Brg1-HIF1α complex as proposed here, may contribute to Chka up-regulation in hepatocytes. Aoyama et al. (2007) have shown that stimulation of Chka transcription by AP-1 is an early event in CCl_4_ induced liver injury and repair. Domizi et al. (2014) have reported that C/EBPβ mediates retinoic acid induced Chka trans-activation in neuronal differentiation. In another report, Gréchez-Cassiau et al. (2015) have demonstrated the Chka transcription in hepatocytes is controlled by the circadian clock *via* the BMAL1-REV-ERBα axis. Of note, Brg1 is known to interact separately with AP-1 (Weng et al., 2015), C/EBPβ (Fan et al., 2019), and ERBα (Zhu et al., 2015). On the other hand, AP-1 (Stepniak et al., 2006), C/EBPβ (Jin et al., 2015), and the circadian clock (Matsuo et al., 2003) each has a well-established role in promoting liver regeneration. It is possible that a multiple-factor complex with Brg1 residing in the center directs the transcription of Chka in proliferating hepatocytes.

One of the better characterized functions of Chka is the biosynthesis of phosphatidylcholine (PC). In the present study, we focused on the transcriptional regulation of Chka in hepatocytes and its implication in the context of liver regeneration. It remains to be determined, however, whether the ability of Chka to contribute to hepatocyte proliferation (Figure 2) is strictly reliant on its function as an enzyme involved in PC production. Of note, CTP:Phosphocholine Cytidylyltransferase (CCT), rather than Chka, is the rate-limiting enzyme in the PC synthesis pathway (Kent, 1997). Tessner et al. (1991) are among the first to report that CCT expression can be stimulated by growth factors in proliferating cells. Houweling et al. (1997) have observed an increase in CCT activity, likely owing to up-regulation of its expression, in the proliferating murine livers. However, targeted deletion of CCT in the liver, as demonstrated by Ling et al. (2012), although indeed leading to impaired PC synthesis does not seem to influence liver regeneration after PHx in mice casting doubts on the causality of PC synthesis in liver regeneration. Thus, it is likely that Chka may regulate hepatocyte proliferation independent of its role in PC synthesis. This hypothesis certainly deserves further investigation.

In summary, our data unveil Chka as a novel transcription target for Brg1 and implicate Chka as a potential regulator of hepatocyte proliferation (Figure 6C). Major limitations of the present study include (1) the lack of direct *in vivo* evidence showing the causal relationship between Chka and liver regeneration, (2) the underlying mechanism whereby Chka contributes to hepatocyte proliferation, and (3) the relatively small sample size of human ALF specimens. Additional investigation, harnessing new transgenic mouse models and transcriptomic analyses, is needed to solidify the role of the Brg1-Chka axis in liver regeneration so that novel therapeutics based on this finding, through screening for small-molecule compound boosting Chka activity/expression, could be devised to treat liver failure.

## Data Availability Statement

The original contributions presented in the study are included in the article/**Supplementary Material**, further inquiries can be directed to the corresponding author/s.

## Ethics Statement

The animal study was reviewed and approved by Nanjing Medical University Ethics Committee on Humane Treatment of Lab Animals.

## Author Contributions

QY, YW, and YX conceived the project. MK, WD, HX, ZF, XM, YG, and CL designed the experiments, performed the experiments, collected data, and analyzed data. YX wrote the manuscript. QY and YW provided funding and supervision. All authors contributed to the article and approved the submitted version.

## Conflict of Interest

The authors declare that the research was conducted in the absence of any commercial or financial relationships that could be construed as a potential conflict of interest.

## Publisher’s Note

All claims expressed in this article are solely those of the authors and do not necessarily represent those of their affiliated organizations, or those of the publisher, the editors and the reviewers. Any product that may be evaluated in this article, or claim that may be made by its manufacturer, is not guaranteed or endorsed by the publisher.
